# Assessing the Potential of an Enzymatically Liberated Salmon Oil to Support Immune Health Recovery from Acute SARS-CoV-2 Infection via Change in the Expression of Cytokine, Chemokine and Interferon-Related Genes

**DOI:** 10.3390/ijms25136917

**Published:** 2024-06-24

**Authors:** Crawford Currie, Tor Åge Myklebust, Christian Bjerknes, Bomi Framroze

**Affiliations:** 1 Hofseth BioCare, Keiser Wilhelms Gate 24, 6003 Alesund, Norway; chbj@hofsethbiocare.no (C.B.); bf@hofsethbiocare.no (B.F.); 2 Department of Research and Innovation, More og Romsdal Hospital Trust, 6026 Ålesund, Norway; tor.age.myklebust@helse-mr.no; 3 Department of Registration, Cancer Registry of Norway, 0379 Oslo, Norway

**Keywords:** COVID-19, long COVID, gene expression, immune health, cytokine, chemokine, interferon, lipid mediators

## Abstract

Cytokines, chemokines, and interferons are released in response to viral infection with the ultimate aim of viral clearance. However, in SARS-CoV-2 infection, there is an imbalanced immune response, with raised cytokine levels but only a limited interferon response with inefficient viral clearance. Furthermore, the inflammatory response can be exaggerated, which risks both acute and chronic sequelae. Several observational studies have suggested a reduced risk of progression to severe COVID-19 in subjects with a higher omega-3 index. However, randomized studies of omega-3 supplementation have failed to replicate this benefit. Omega-3 fats provide important anti-inflammatory effects; however, fatty fish contains many other fatty acids that provide health benefits distinct from omega-3. Therefore, the immune health benefit of whole salmon oil (SO) was assessed in adults with mild to moderate COVID-19. Eleven subjects were randomized to best supportive care (BSC) with or without a full spectrum, enzymatically liberated SO, dosed at 4g daily, for twenty-eight days. Nasal swabs were taken to measure the change in gene expression of markers of immune response and showed that the SO provided both broad inflammation-resolving effects and improved interferon response. The results also suggest improved lung barrier function and enhanced immune memory, although the clinical relevance needs to be assessed in longer-duration studies. In conclusion, the salmon oil was well tolerated and provided broad inflammation-resolving effects, indicating a potential to enhance immune health.

## 1. Introduction

The successful development of pharmaceutical interventions for SARS-CoV-2 infection has dramatically reduced the associated morbidity and mortality of COVID-19. Nevertheless, COVID-19 continues to impose significant health challenges, not least from the post-acute sequelae of COVID-19 (PASC), commonly referred to as long COVID.

Marked elevations in the pro-inflammatory cytokines IL-6 and TNFα, predominantly secreted by lung macrophages, appear central to both the effects of acute infection and long COVID [1,2]. Serum CXCL-10, which activates macrophages via the CXCR3 receptor, is also typically elevated and further increases the inflammatory response. Higher CXCL-10 levels are also associated with an increased risk of long COVID [3,4].

Although the lungs are the first organ to be affected by SARS-CoV-2 infection, the virus can directly infect many organs, including the gut, kidney, brain, myocardium, and vasculature. This is likely a significant driver of the common long-term sequelae of COVID-19, such as shortness of breath, fatigue, brain fog, and sleep disturbances [5,6].

SARS-CoV-2′s ability to evade recognition by the innate immune system results in lower levels of interferon-I and -III in the lungs and peripheral blood compared to respiratory viral infections [7]. The innate immune system uses pattern recognition receptors (PRRs) to detect the presence of viruses and mount an immune response to clear the virus (8). This detection system includes toll-like receptors (TLRs) and ultimately results in the release of inflammatory cytokines and chemokines, type I and III interferons, and the activation of immune effector cells, especially natural killer (NK) cells [8,9,10]. In contrast, COVID-19 infection results in an imbalanced response with a significant cytokine response but a muted interferon response and hence reduced cellular antiviral activity. Compounding this further is an increase of TGFβ seen with SARS-CoV-2 infection, which reduces NK cell anti-viral activity and viral clearance [11].

However, whilst viral persistence is observed in up to 50% of long COVID sufferers, in others, the virus is undetectable but the inflammatory response, characterized by IL-6 and TNFα, persists [12]. Immune dysregulation with elevated inflammatory markers therefore appears central to the pathology and symptoms of long COVID [13,14].

The management of long COVID remains a significant challenge, with estimates of at least 65 million individuals worldwide affected by this condition [13]. Furthermore, whilst the absolute risk of long COVID is lower in those suffering mild infections, this cohort represents the largest single group of COVID-19 infections and, therefore, a significant contributor to long COVID cases. Indeed, COVID-19 results in a prolonged inflammatory signature even after apparent recovery from acute infection.

Observational data, including from the United Kingdom Biobank, has suggested that subjects with a higher omega-3 index (O3i) have a lower risk of hospitalization and severe COVID-19 [15]. The regular consumption of fresh fish provides anti-inflammatory and antioxidant benefits from the fatty fraction of the fish. The metabolism of omega-3 fatty acids results in the production of specialist pro-resolving mediators (SPMs) of inflammation [16]. However, other fractions within fish oil appear to provide anti-inflammatory benefits distinct from omega-3. These effects include the regulation of nuclear factor-kappa beta (NF-kB) activation and the moderation of inflammatory responses, including the reduced expression of the pro-inflammatory cytokines IL-6 and IL-8 [17,18,19]. A non-fatty fraction of microcolin lipopeptides, derived from fish-eating blue-green algae, has also been described as moderating type II inflammation by modulating eosinophil effector function [20]. These wide-ranging inflammation-resolving effects of marine fatty acids might, therefore, explain how subjects with higher O3i levels were able to fare better when infected by COVID-19.

OmeGo is enzymatically liberated from Norwegian Atlantic salmon and has the same lipid profile as contained in whole salmon. Our hypothesis was that OmeGo might, therefore, aid recovery from acute COVID-19 infection and thereby reduce the risk of progression to severe disease. To this end, our study recruited patients with milder forms of COVID-19 and subjects were randomized to receive standard-of-care treatment with or without OmeGo. This study assessed both clinical and biomarker outcomes. The biomarker work, assessing the immune response to COVID-19 infection and recovery along with biomarkers associated with long COVID, is the focus of this paper.

## 2. Results

This study ran from 10 August 2021 to 19 May 2022. The gene expression analysis set was comprised of eleven subjects, six in the OmeGo group and five in the BSC group. Four of the six subjects in the OmeGo group were female and two were male, with an average age of 33.8 years, and in the BSC group, three were female and two were male, with an average age of 29.4 years (Table 1). As noted, nasal swabs were taken on days zero, fourteen, and twenty-eight.

The two groups were balanced in terms of sex, age, and BMI, whereas there was an imbalance in terms of ethnicity. Mean blood leukocyte count, CRP, and glucose were all normal at the baseline and on day twenty-eight, as were blood electrolytes, creatinine, and liver enzymes. There were no statistically significant differences between treatment arms on any clinical endpoints. No serious adverse events were reported during this study. Overall, two subjects (one in the BSC group and one in the OmeGo group) reported three adverse events that were judged not to be related to the study treatment.

### 2.1. Change in Cytokine Gene Expression Levels

**Figure 1:**
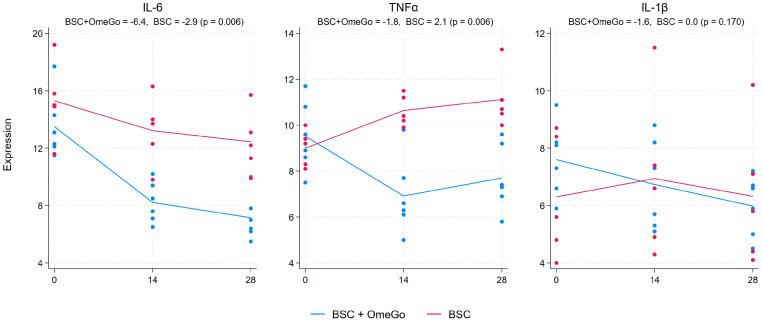
Fold change in the gene expression of cytokines IL-6, TNFα, and IL-1β from the baseline to day twenty-eight. The numeric change from day zero to day twenty-eight is given for both groups at the top of the chart along with the *p*-value to assess for significance of the difference between the two groups. The significance level was set at 5% (*p* ≤ 0.05). The blue lines represent the BSC and OmeGo groups and the red lines the BSC-only group. The dots represent the gene expression level for the individual subjects. The lines represent a line of best fit and do not go through the mean scores.

The gene expression analysis focused on the cytokines closely associated with acute SARS-CoV-2 infection and recovery and those associated with long COVID. The gene expression of the core pro-inflammatory cytokines in COVID-19, IL-6, and TNFα, were significantly lower with OmeGo than in the BSC group (*p* < 0.01). The expression of IL-6 halved from the baseline with OmeGo, whereas it declined by around 25% in the BSC group, resulting in an inter-group difference of three fold on day 28 (Figure 1). TNFα expression was reduced by around 20% with OmeGo, whereas it increased by almost 25% in the BSC group, resulting in a 3.4-fold difference by the end of this study (Figure 1). Elevations of IL-1β are also noted in both acute and long COVID, as well as IL-8, IL-13, GM-CSF (granulocyte–monocyte stimulatory factor), and TGFβ. In our study, IL-1β expression was just over 20% higher in the OmeGo subjects at the baseline compared to the BSC group, and whilst there was a 1.6-fold decline in IL-1β with OmeGo (and no change in the BSC arm), there was ultimately no difference between the two arms at the end of this study (Figure 1).

**Figure 2:**
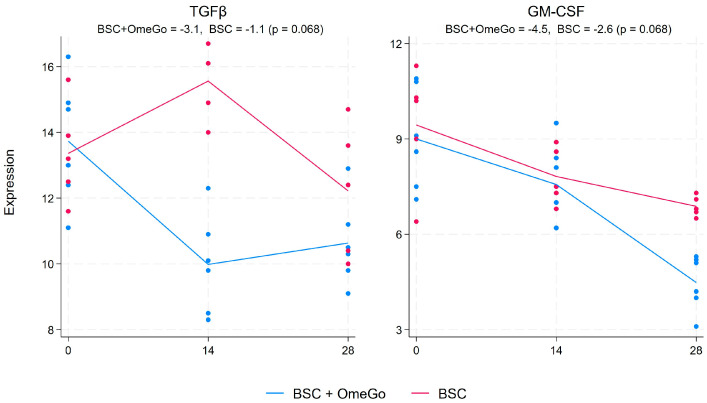
Fold change in the gene expression of TGFβ and GM-CSF from the baseline to day twenty-eight. The numeric change is given for both groups at the top of the chart along with the *p*-value to assess for significance of the difference between the two groups. The significance level was set at 5% (*p* ≤ 0.05). The blue lines represent the BSC and OmeGo groups and the red lines the BSC-only group. The dots represent the gene expression level for the individual subjects. The lines represent a line of best fit and do not go through the mean scores.

There was also no difference seen in IL-8, with expression levels unchanged over the duration of the trial in either arm. In contrast, IL-13 expression was reduced by over 40% with OmeGo, whilst it increased by 25% in the BSC group, resulting in a 4-fold difference on day 28 (*p* < 0.01). The expression of IL-4 remained unchanged with BSC and showed a limited 0.9-fold increase with OmeGo, which was significant (*p* < 0.01). GM-CSF also declined more in the OmeGo arm; however, the 2.4-fold difference was not significant, and a similar outturn was seen with TGFβ, with a 1.6-fold difference in favor of OmeGo, which again was non-significant (*p* = 0.07 for both) (Figure 2).

### 2.2. Change in Chemokine Gene Expression Levels

**Figure 3:**
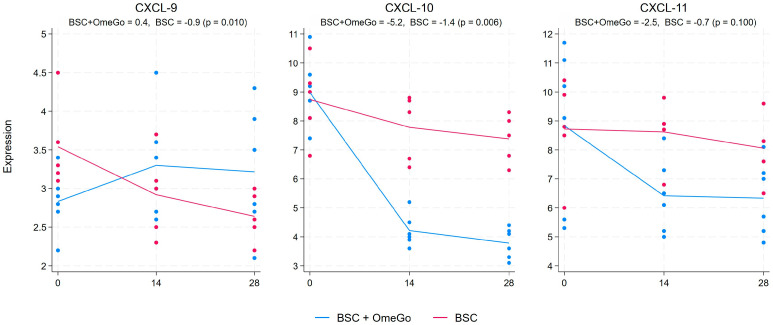
Fold change in the gene expression of chemokines CXCL-9, CXCL-10, and CXCL-11 from the baseline to day twenty-eight. The numeric change is given for both groups at the top of the chart along with the *p*-value to assess for significance of the difference between the two groups. The significance level was set at 5% (*p* ≤ 0.05). The blue lines represent the BSC and OmeGo groups and the red lines the BSC-only group. The dots represent the gene expression level for the individual subjects. The lines represent a line of best fit and do not go through the mean scores.

**Figure 4:**
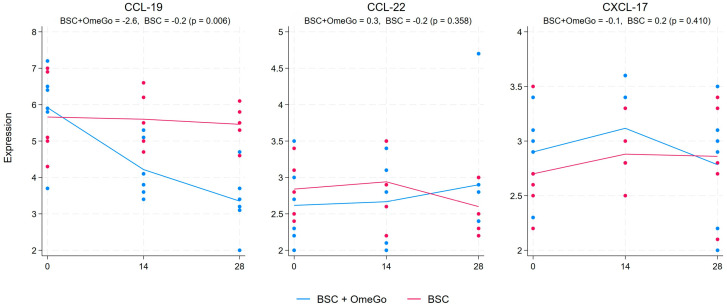
Fold change in the gene expression of chemokines CCL-19, CCL-22, and CXCL-17 from the baseline to day twenty-eight. The numeric change is given for both groups at the top of the chart along with the *p*-value to assess for significance of the difference between the two groups. The significance level was set at 5% (*p* ≤ 0.05). The blue lines represent the BSC and OmeGo groups and the red lines the BSC-only group. The dots represent the gene expression level for the individual subjects. The lines represent a line of best fit and do not go through the mean scores.

**Figure 5:**
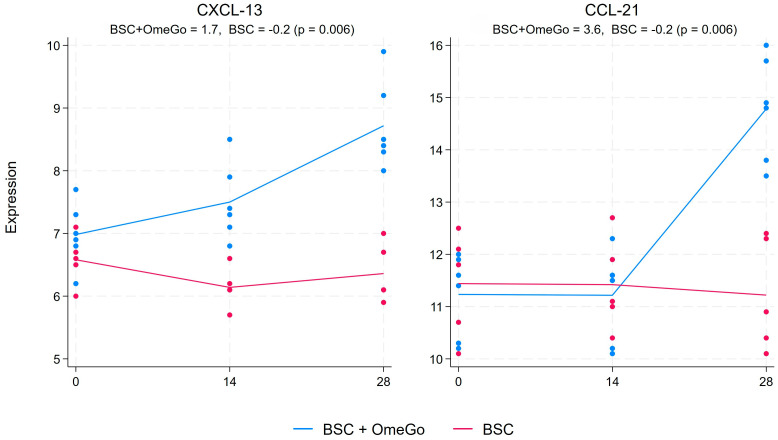
Fold change in the gene expression of chemokines CXCL-13 and CCL-21 from the baseline to day twenty-eight. The numeric change is given for both groups at the top of the chart along with the *p*-value to assess for significance of the difference between the two groups. The significance level was set at 5% (*p* ≤ 0.05). The blue lines represent the BSC and OmeGo groups and the red lines the BSC-only group. The dots represent the gene expression level for the individual subjects. The lines represent a line of best fit and do not go through the mean scores.

The chemokine analysis showed a more than halving of CXCL-10 expression from the baseline to the end of this study in the OmeGo arm, with a small decline in the control arm, which resulted in an almost four-fold significant difference in expression between the two arms (*p* < 0.01). The associated chemokines, CXCL-9 and CXCL-11, showed more limited changes (Figure 3). CXCL-9 trended up in the OmeGo arm and down in the control arm, resulting in a 0.6-fold difference (*p* = 0.01), whereas the reduction in CXCL-11 with an almost 2-fold expression difference compared to the control missed significance (*p* = 0.1). CCL-19 showed a significant 2-fold greater reduction in expression with OmeGo, and levels in the control group were basically unchanged (*p* < 0.01). CCL-22 and CXCL-17, which are often raised alongside CCL-19 in COVID-19, showed little difference between the two arms (Figure 4). Changes in the expression of CCL-3 and CCL-5, chemokines associated with acute viral infection, were non-significant and showed little difference between the two arms. Finally, CXCL-13 and CCL-21, chemokines associated with immune memory, were significantly increased with OmeGo by 2.4 and 3.6 folds, respectively (both *p* < 0.01) (Figure 5).

### 2.3. Change in Interferon and Interferon-Related Factors Gene Expression Levels

**Figure 6:**
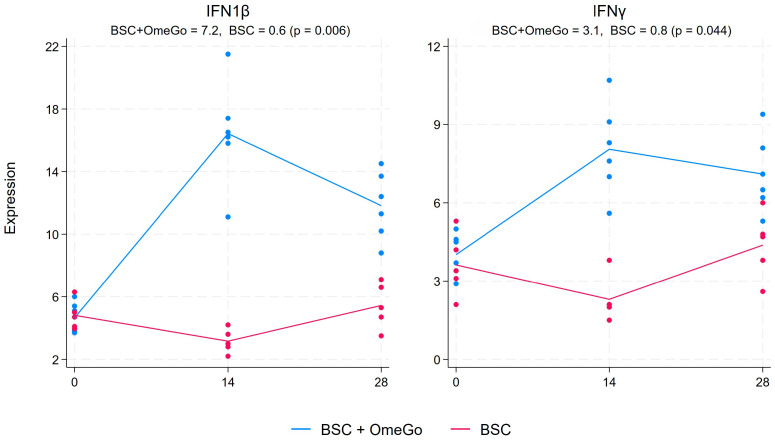
Fold change in the gene expression of interferons IFN1β and IFNγ from the baseline to day twenty-eight. The numeric change is given for both groups at the top of the chart along with the *p*-value to assess for significance of the difference between the two groups. The significance level was set at 5% (*p* ≤ 0.05). The blue lines represent the BSC and OmeGo groups and the red lines the BSC-only group. The dots represent the gene expression level for the individual subjects. The lines represent a line of best fit and do not go through the mean scores.

**Figure 7:**
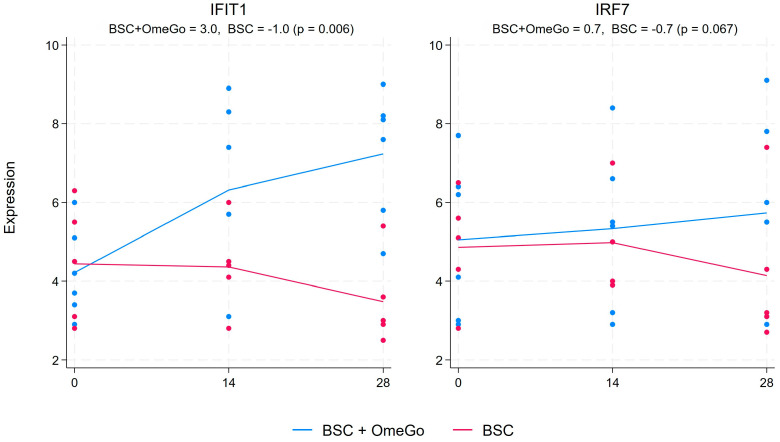
Fold change in the gene expression of the interferon-related factors, IFIT1 and IRF7, from the baseline to day twenty-eight. The numeric change is given for both groups at the top of the chart along with the *p*-value to assess for significance of the difference between the two groups. The significance level was set at 5% (*p* ≤ 0.05). The blue lines represent the BSC and OmeGo groups and the red lines the BSC-only group. The dots represent the gene expression level for the individual subjects. The lines represent a line of best fit and do not go through the mean scores.

In contrast to pro-inflammatory cytokine and chemokine elevations observed in COVID-19, interferon and interferon-related factors are typically under-expressed compared to responses typically seen with other viral infections. OmeGo resulted in a more than doubling of the expression of IFN1β compared to little change in the control arm, with a 6.4-fold difference between the two arms on day 28 (*p* < 0.01). Interestingly, the difference was even more marked on day 14, at which point the expression in the control arm had declined by 1.6 fold, whereas it had increased in the OmeGo arm by almost 12 fold. A very similar pattern was also seen with the expression of IFNγ, with the largest difference between the arms on day 14 and a six-fold difference declining to an almost three-fold significant difference (*p* < 0.05) (Figure 6). The interferon-stimulated gene, IFIT1, showed a sustained increase throughout the study period in the OmeGo group, with an almost 4-fold difference on day 28 versus BSC (*p* < 0.01), whereas the 1.6-fold difference in interferon regulatory factor 7 (IRF7) on day 28 did not reach significance (*p* = 0.07) (Figure 7).

## 3. Discussion

This twenty-eight-day study analyzed changes in gene expression in the nasal mucosa of subjects with milder forms of COVID-19 to assess the impact of OmeGo, an enzymatically liberated salmon oil, on the immune response and recovery from SARS-CoV-2 infection. The subjects were all ambulatory with mild symptoms and, therefore, the response to SARS-CoV-2 infection in the nasal epithelium was proposed as a more sensitive means to assess immune response compared to serum markers. Whilst this is a relatively short-duration study, the twenty-eight-day timeframe does cover the critical time points of the period of peak viral load, the risk period for hospitalization, and the average time to viral clearance with SARS-CoV-2 infection [21,22,23]. Hence, this enabled the assessment of both the initial immune response and early post-infection recovery along with insights into the potential to moderate the risk of long COVID, a syndrome that has been associated with a prolonged inflammatory profile post-SARS-CoV-2 infection [24].

A hallmark of early COVID-19 is an increase in pro-inflammatory cytokines, especially IL-6 and TNFα, as well as IL-1 β, IL-8, GM-CSF, and the type 2 inflammatory cytokine IL-13 [1,25,26]. Whilst such a response is important initially to help control viral replication and spread, excessive or prolonged levels of inflammation can result in damage to the body and ill health.

By day fourteen, subjects in the OmeGo arm showed marked reductions in the expression of IL-6 and TNFα, as well as IL-13, compared to BSC, and this difference persisted to day twenty-eight, and these were all statistically significant. However, the 2.4-fold lower expression of GM-CSF seen with OmeGo did not reach significance, which was perhaps a result of the small number of subjects in our study. IL-1β and IL-8 showed little change in either group. Whilst IL-4 showed a 75% significant relative increase compared to the control, the absolute 0.9-fold difference in expression would question any benefit in terms of reducing the risk of long COVID. Indeed, a minimum fold change of 1.5 is frequently taken as physiologically relevant [27,28].

Chemokines are also central to the early immune response to infection, helping to recruit immune cells to infected areas. Stimulatory loops between cytokines and chemokines help ramp up inflammation and viral clearance. However, as per cytokines, a balanced response with progressive resolution is required for optimal recovery from the infection.

There was an almost 50% greater decrease in the gene expression of the chemokine CXCL-10, an important driver of inflammation in COVID-19, in the OmeGo-treated group [29]. The chemokines, CXCL-9, -10, and -11, are closely related and act through a common receptor, CXCR3, expressed on a number of immune cells including monocytes, T-cells, and NK cells. CXCL-9 and -11 have also been noted to be raised with SARS-CoV-2 infection [30]. OmeGo appeared to have a more limited impact on both of these, and whilst the 1.3-fold increase of CXCL-9 compared to BSC was statistically significant, the physiological impact would likely be limited. CCL-19, CCL-22, and CXCL-17 are also frequently raised in acute COVID-19 cases and are involved in T-cell, dendritic cell, and macrophage chemotaxis. CCL-19 showed a significant 2-fold greater reduction in expression with OmeGo with levels basically unchanged in the control group, and minimal changes were seen with CCL-22 and CXCL-17 in either group of subjects. Limited changes were seen in the expression of CCL-3 and CCL-5, chemokines that are often raised in viral infections and help recruit both NK cells and macrophages. Finally, the significant increases in CXCL-13 and CCL-21 are noteworthy. These play a part in antigen-specific B-cell maturation, T-cell activation, and immune self-tolerance. Whether this suggests a potential for a more sustained immunity against SARS-CoV-2 is unclear as we did not assess other markers, such as the expression of CD27 and CD21 on B-cells and plasma cells, to provide greater weight to this potential effect [31,32]. Furthermore, excessive amounts of CXCL-13 have been associated with worse outcomes in COVID-19 [33]. As always, a balance of activity is required with the immune system to support ongoing health.

Taken together, these cytokine and chemokine changes could suggest that OmeGo helped restore a healthier level of inflammation compared to the control. IL-6 and TNFα are central to the inflammatory response in COVID-19, and IL-13 has also been linked to disease severity, driving increased levels of eosinophils and macrophages in the lungs and stimulating smooth muscle hypertrophy and fibrosis [1,25,26,27]. CXCL-10 is associated with IL-6 secretion with raised levels seen in COVID-19 patients with pulmonary immune cell infiltration and cytokine storm [29]. The 2.4-fold reduction in GM-CSF is consistent with a decreased inflammatory profile, with GM-CSF being involved in the differentiation of alveolar macrophages and changes in lung barrier function [26]. As such, this reduction might suggest a faster resolution of lung inflammation; however, the change versus BSC did not reach statistical significance. Finally, whilst the three-fold decline from the baseline in TGFβ with OmeGo is consistent with a potentially improved immune profile, the 1.6-fold greater decrease in TGFβ versus BSC missed statistical significance.

In contrast to the inflammatory cytokine response to acute SARS-CoV-2 infection, limited interferon production can blunt the overall immune response with an increased risk of severe disease. This effect is linked to the production of inhibitory proteins encoded by SARS-CoV-2 [6,34,35]. Subjects in the OmeGo arm showed a significant, greater than six-fold increase in interferon-1 (IFNβ1). Type 1 interferon response is typically viewed as helping drive the most robust anti-viral immune response and helping to improve barrier function in the lungs and gut, along with type III interferons [9,36,37,38].

Treatment with OmeGo also resulted in a near doubling expression of IFNγ (type II interferon) and a significant difference compared to the limited change in the BSC arm. Whilst IFNγ is not typically involved in the natural course of SARS-CoV-2 infection, it does appear to augment the immune response against SARS-CoV-2 infection in the lungs. A study of IFNγ in almost 2000 outpatients presenting with COVID-19 within seven days of infection showed a halving of subsequent hospitalization compared to those receiving a placebo despite high vaccination rates [39]. Further, a study in hospitalized patients with moderate, new SARS-CoV-2 infection showed a faster recovery in those receiving IFNγ; however, a small case series in critically ill patients did not show a benefit for IFNγ [40,41]. In total, these studies suggest that the greatest impact of IFNγ on COVID-19 is in the earlier stages of infection. Consistent with this, subjects who suffered from respiratory infections prior to contracting SARS-CoV-2 seem to have a more robust immune response (including an IFNγ response), resulting in milder COVID-19 [42].

The interferon response in this study for both IFN1β and IFNγ peaked on day 14 and progressively declined from there onwards. This might suggest that OmeGo helped support a better interferon response; however, we did not assess the impact on viral clearance or longer-term outcomes, such as long COVID. We did, however, see consistent effects in the interferon-related immune system. The action of interferon includes the induction of interferon-stimulated genes (ISGs), which target different parts of the viral replication cycle [43]. Interferon regulatory factors (IRFs) also induce the expression of ISGs in response to TLR (toll-like receptors) and RLR (RIG-1-like receptors) activation. One important group of ISGs is the IFIT proteins (interferon-induced protein with tetratricopeptide repeats), which target viral protein production [44]. IFIT1 is a central part of this defense mechanism, and a muted IFIT1 response is seen in SARS-CoV-2 infection, which is probably a result of direct viral mechanisms and a limited interferon response. Mirroring the increase seen with IFN-1β and -γ, OmeGo showed a significant two-fold increase in IFIT1 expression. Whilst this could suggest a potential for more efficient clearing of the viral infection, further work is needed to explore this potential benefit.

The increase in the gene expression of IRF7 (interferon-regulatory factor-7) was less marked at 1.6 fold compared to BSC, and this change missed significance. Interferon regulatory factors (IRFs) help sustain the production of type I and III interferons and help reduce their expression after viral clearance, and IRF7 is considered a key IRF [45,46,47]. The limited change in IRF7 could reflect the relatively mild cases of COVID-19 in this study, which would not require prolonged IFN production. Consistent with this, the expression of both IFNβ and IFNγ declined by 50% from day 14 to day 28, which suggests a resolving infection.

Overall, these changes suggest that the subjects in the OmeGo arm had a better immune health profile by day 28. The interferon response appears improved and is combined with a progressive reduction in the expression of pro-inflammatory cytokines and chemokines. Even in mild cases of COVID-19, the immune inflammatory gene signature can remain exaggerated for many months after the resolution of symptoms [48]. Monocyte-derived macrophages continue to express a number of inflammatory genes, in particular, lipid mediators of immune function, which is, in essence, an upregulation of eicosanoid (prostaglandins, leukotrienes, and thromboxane) production with a downregulation of lipid mediators involved in inflammation resolution [48,49,50].

Lipid mediators are an important part of the immune response. Eicosanoids, metabolites of 5-lipoxygenase, have numerous actions, including white cell chemotaxis, as well as increasing vascular permeability to enable immune cells to reach infected areas. They also help sustain the immune response. However disordered lipid metabolism with increased eicosanoid production and reduced SPM expression can result in fibrosis with organ damage, including airway remodeling and reduced lung function. Raised IL-6 and TNFα levels appear central to PASC/long COVID and complications, such as the increased risk of cardiovascular events, persist long after the acute infection, as does the neuroinflammation and associated cognitive deficits (“brain fog”) [51,52]. IL-1, IL-13, CXCL-10, and TGFβ are also raised in long COVID, especially in those with respiratory complications and suppressed levels of IL-4 and IL-10 [1,2,53].

OmeGo not only contains the omega-3 polyunsaturated fatty acids eicosapentaenoic acid (EPA), docosapentaenoic acid (DHA), and docosahexaenoic acid (DPA) but also omega-5, omega-6, omega-7, omega-9, and omega-11. The health benefits of omega-3 fatty acids derive from their metabolism into three different classes of SPMs, namely, protectins, resolvins, and maresins, which support immune and overall health [54]. For instance, specialized pro-resolving mediators (SPMs) reduce the production of inflammatory cytokines, including IL-6, modify macrophage function to a more protective role, and balance neutrophil and T-cell activity with other SPMs, appearing to suppress viral replication [55,56,57].

These combined effects could enhance recovery from viral infections, consistent with the observation of a higher omega-3 index appearing protective against severe COVID-19 [17]. However, studies of omega-3 supplements have delivered somewhat equivocal results. A large Norwegian study of cod liver oil (containing EPA, DHA, and vitamin D) showed no benefit in preventing COVID-19 or reducing its severity and, overall, other clinical trials have not shown a conclusive benefit for omega-3 oils containing EPA and DHA in COVID-19 [58,59].

The importance of DPA in immune health has emerged more recently than that of EPA and DHA, and DPA is now recognized as the source of a distinct family of SPMs. These SPMs have a range of actions, including the inhibition of viral replication and reduced platelet aggregation [57,60,61,62]. Recent publications have also shown the health benefits of non-omega-3 fatty acids contained in fish oil. For example, omega-9 (oleic acid) and pentadecanoic acid provide non-overlapping anti-inflammatory effects with cardiometabolic health benefits [63,64]. For oleic acid, at least part of this effect appears to be via a reduction in the inflammatory action of advanced glycation end products (AGEs) by raising levels of soluble receptors for advanced glycation end products (sRAGEs), a decoy receptor that has no transcriptional activity [65]. This reduces the AGEs available to bind to membrane RAGE (mRAGE) to trigger cellular inflammation and oxidative stress. Lower levels of sRAGE occur with aging and in chronic diseases, such as obesity, and have been correlated to COVID-19 severity and also implicated in long COVID [66].

The array of polyunsaturated and monounsaturated fats contained in OmeGo could, therefore, provide broader inflammation-resolving and immune health benefits than an intervention focused on two omega-3 fatty acids, namely, EPA and DHA, and more closely mimic the health benefits of regularly eating whole fish. Beyond the anti-inflammatory effects of oleic acid and pentadecanoic acid noted above, OmeGo also contains a lipopeptide fraction composed of microcolins. These originate from blue-green algae, a natural part of the fish’s diet, and have been shown to moderate type 2 eosinophilic inflammation, including a reduction in eosinophilic activation and migration and associated reductions in IL-13 levels [20,67,68].

We did not measure plasma cytokine levels and note that changes in mRNA expression and protein levels can show a variable correlation due to various biological factors, such as post-translational modifications. The overall correlation between mRNA and protein expression is generally positive but moderate, as observed in similar studies of human monocytes [69]. Correlations likely depend on the gene categories in question, as the transcription and translation of various genes will be variably modified prior to the final protein synthesis. Quantification methods employed also have some inherent variability, adding some noise to the correlation. The strength of the correlations also appears context dependent. For instance, the differential expression of mRNA versus the control shows better correlations with their protein products compared to non-differentially expressed ones. This would inherently make sense that a differential mRNA expression should have a functional impact, such as a change in protein expression. If not, it would question the purpose of the differential expression. This was demonstrated in an ovarian cancer xenograft model, where differentially expressed mRNAs showed significant protein correlation, increasing confidence in using mRNA data for biological discovery in specific contexts [70]. The generally positive strength of the correlations was the basis for investigating differential mRNA expression in this research.

Our study has some weaknesses. We only followed the subjects up for twenty-eight days, and we have no data on whether subjects in either group subsequently suffered from long COVID. Nevertheless, this time period does include important time points relevant to acute infection and recovery. It should also be noted that the biomarker study was a sub-study of only eleven subjects with an average age of 32 years and only two subjects in their sixth decade. The effectiveness of the immune system against infectious diseases, including COVID-19, declines with age. A nutritional supplement to better support immune health in the elderly would be an important finding; however, our study does not provide any insights into elderly subjects. We did not measure the change in viral load, which would have further supported the immune health benefits indicated by the changes in gene expression seen in this study. Delayed viral clearance has been correlated to higher levels of TNFα and IL-6 and lower levels of neutralizing antibodies with a greater risk of severe disease and long COVID [51,52,71]. Whilst we assessed the change in TNFα and IL-6 expression, we did not assess blood antibody levels, which would have strengthened the data into a potential for OmeGo to support efficient viral clearance. Finally, we did not measure the change in the omega-3 index, which could have provided further insights into the relative drivers of inflammation resolution with OmeGo. Nevertheless, the biomarker changes seen in the OmeGo group are consistent with previously published research, both with OmeGo and on the modulation of immune signaling by lipid mediators. Further clinical trial work is needed to delineate OmeGo’s potential role in supporting immune health, such as the prevention or treatment of long COVID, a condition of immune dysregulation and inflammation. Pollution-related cough, resulting from underlying particulate matter inflammation, is another area of unmet need with a significant impact on quality of life, including sleep quality. It also has a broad inflammatory signature with similarities to that seen in COVID-19, albeit at a significantly lower level. A low-cost intervention derived from fresh fish would be an appealing approach for this common symptom, and clinical trials should be considered in this setting.

## 4. Materials and Methods

### 4.1. Study Design and Study Subjects

This randomized, open-label, parallel study was conducted in five sites in three countries: Hungary, Serbia, and Brazil. It was designed by the sponsor, Hofseth BioCare, with input from Eurofins (Luxembourg City, Luxembourg), the Clinical Research Organization that managed the trial sites. Institutional review boards approved this study at each site, and it was run in accordance with the principles of the Declaration of Helsinki, International Conference on Harmonization (ICH) guidelines for current Good Clinical Practice (GCP), and applicable regulatory requirements. All subjects freely provided their written, informed consent.

**Figure 8:**
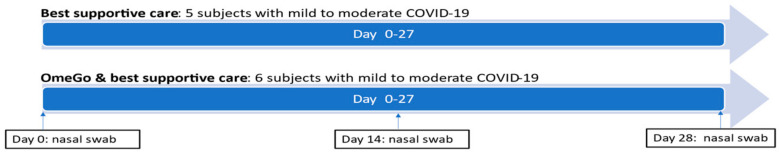
Trial design. Subjects with mild to moderate COVID-19 infection were randomized to best supportive care (BSC) with or without OmeGo for 28 days. Voluntary nasal swabs to analyze changes in the gene expression of markers of immune response and recovery were taken on days zero, fourteen, and twenty-eight.

Subjects were eligible if they were aged between 18 and 75 years with confirmed mild to moderate COVID-19 infection. Patients with a known fish allergy or hypersensitivity were excluded. Enrolled patients were randomized on day 0 to either OmeGo plus the best standard of care (BSC) or BSC alone. Patients randomized to the OmeGo arm were instructed to take two capsules (equivalent to 2 g of salmon oil) twice daily. They were also requested to save all unused and open packages to determine treatment compliance. A standard assessment, including physical, laboratory, and radiographic investigations, was undertaken to assess the patient’s clinical condition at the baseline, and the same assessments were undertaken periodically through the twenty-eight days of this study. On days zero, fourteen, and twenty-eight, nasal swabs were taken for gene expression analysis (Figure 8). The selected genes were those involved in the immune response to COVID-19 infection, recovery from COVID-19, and markers associated with long COVID.

### 4.2. Gene Expression Analysis

Following the collection of the nasal swab samples, each swab was vortexed while still in the tube with the transport medium for fifteen seconds. Using sterile forceps, the swab was removed from the transport tube and squeezed by pressing it against the side of the tube to wring out any remaining fluid and to ensure that most of the cells were in the liquid medium.

The RNeasy UCP Micro Kit (Qiagen, Venlo, Netherlands) and the standard protocol were used to purify mRNA from the nasal epithelial cells. The fluid collected from each of the 3 × 11 patient swabs was pelleted by centrifugation for five minutes at 1000 rpm in a centrifuge tube. All the supernatant was carefully removed by aspiration, making sure that all the cell media had been removed thoroughly. The cells were disrupted by adding a 350 µL buffer RULT, taking care to loosen the cell pellet from the tube, vortexed to mix thoroughly, and the mixture was homogenized by passing the lysate five times through a 20-gauge needle fitted to an RNase-free syringe. Next, 350 µL of 70% ethanol was added to the lysate and mixed again by pipetting (some precipitate was visible but was not a problem for the assays).

The sample was transferred, including any precipitate that may have formed, to an RNeasy UCP MinElute spin column placed in a 2 mL collection tube, centrifuged for fifteen seconds at 10,000 rpm, and washed as per the instructions. Next, 10 µL DNase I stock solution was added to 70 µL buffered RDD, mixed gently, and 80 µL of the DNase I mix was added directly to the spin column membrane and vortexed for 15 min. Then, 350 L buffer RUWT was added to the spin column and centrifuged for 15 s at 10,000 rpm to wash it, and the flow through was discarded. The spin column was placed in a new 2 mL collection tube, 500 µL of buffer RUPE was added and centrifuged for fifteen seconds at 10,000 rpm, and the flow through was discarded.

The spin column was washed again with 500 µL of 80% ethanol, placed in a new 2 mL collection tube, and centrifuged for 5 min at full speed with the lid open to make sure all the ethanol was removed. The spin column was placed in a new 1.5 mL collection tube, 14 µL ultra-clean water was added directly to the center of the spin column membrane, the lid was closed, and it was centrifuged for 1 min at full speed to elute the RNA. The dead volume of the spin column was 4 µL, which was eluted with 16 µL of ultra-clean water to yield a ~20 µL (4 µg) RNA eluate.

For cDNA preparation for RT-PCR, the RNA samples from each of the 3 × 11 patients were added to 40 µL of buffer GE2 (gDNA elimination buffer) and RNase-free water to make a final volume of 60 µL. This was incubated at 37 °C for five minutes and immediately placed on ice for two minutes, and 62 µL of the BC5 Reverse Transcriptase Mix was added to each 60 µL RNA sample for a final volume of 102 µL. This was incubated at 42 °C for exactly fifteen minutes, and then the reaction was immediately stopped by heating at 95 °C for five minutes (then held on ice until qPCR as required).

The Human Oxidative Stress RT2 Profiler PCR Array (Qiagen, Venlo, Netherlands) was used, as RT-PCR is a highly sensitive and reliable method for gene expression analysis. The assay was used to analyze expression levels of nineteen genes related to the immune response in nasal epithelial cells from SARS-CoV-2-positive subjects treated with and without CARDIO salmon oil capsules for twenty-eight days. The cDNA from above was mixed with the RT2 SYBR Green fluor Mastermix (Qiagen, Venlo, Netherlands) and aliquoted into the wells of the PCR Array. RT-PCR was performed on an iCycler (Bio-Rad Labs, Hercules, CA, USA). Gene expression was compared using Ct values, and the results were calculated using the ΔΔ Ct. method with normalization to the average expression levels of the five common genes (ACTB, B2M, GAPDH, HPRT, and RPL13A).

## 5. Conclusions

In summary, in subjects with milder forms of SARS-CoV-2 infection, twenty-eight days of treatment with OmeGo, an enzymatically liberated whole salmon oil, resulted in changes in the cytokine, chemokine, and interferon gene expression, which suggests potential for a faster return to immune homeostasis. This is consistent with the published literature on the inflammation-resolving effects of the range of lipid mediators contained in whole fish. Further clinical trials are needed to assess how this translates into outcomes, such as reducing the complications of infection, such as long COVID.

## Figures and Tables

**Table 1:** Age distribution and ethnicity of the study subjects. Southeast Asian denotes persons having origin from any of the following countries: Thailand, Singapore, Indonesia, and Taiwan.

Age Range (Years)	Number of Subjects	Subjects on OmeGo and BSC	Subjects on BSC Only
20–29	6	2	4
30–39	3	3	0
40–49	0	0	0
50–59	2	1	1
Mean age (years)		33.8	29.4
Ethnicity			
Caucasian		6	3
Southeast Asian		0	2

## Data Availability

Data requests can be directed to the authors of this paper.
